# Mucolipidosis Type IV in Omani Families with a Novel *MCOLN1* Mutation: Search for Evidence of Founder Effect

**DOI:** 10.3390/genes13020248

**Published:** 2022-01-28

**Authors:** Badriya Al-Alawi, Beena Harikrishna, Khalid Al-Thihli, Sana Al Zuhabi, Anuradha Ganesh, Zainab Al Hashami, Zeyana Al Dhamhmani, Razan Zadjali, Nafila B. Al Riyami, Fahad Zadjali

**Affiliations:** 1 Clinical Biochemistry Program, Oman Medical Specialty Board, Muscat 123, Oman; badriyaalawi@gmail.com; 2 Department of Ophthalmology, Sultan Qaboos University Hospital, Muscat 123, Oman; beena.harikrishna@gmail.com (B.H.); zuhaibi@hotmail.com (S.A.Z.); gananu@gmail.com (A.G.); 3 Department of Genetics, Sultan Qaboos University Hospital, Muscat 123, Oman; khalid.althihli@gmail.com; 4 Department of Clinical Biochemistry, College of Medicine and Health Sciences, Sultan Qaboos University, Muscat 123, Oman; alhashami@squ.edu.om (Z.A.H.); aldahmani@squ.edu.om (Z.A.D.); razanzadjalii@gmail.com (R.Z.); nafila@squ.edu.om (N.B.A.R.)

**Keywords:** *MCOLN1*, Oman, mucolipidosis, retinal dystrophy, corneal clouding, mendelian inheritance

## Abstract

Mucolipidosis Type IV (MLIV) is caused by a deficiency of the mucolipin cation channel encoded by Mucolipin TRP Cation Channel 1 gene (*MCOLN1*). It is a slowly progressive neurodevelopmental and neurodegenerative disorder causing severe psychomotor developmental delay and progressive visual impairment, which is often misdiagnosed as cerebral palsy. We describe six patients with MLIV from two Omani families with a novel c.237+5G>A mutation in the *MCOLN1* gene predicted to affect mRNA splicing. Mutation screening with a high-resolution melting (HRM) assay in a large population sample did not detect this mutation in control subjects. This report highlights the importance of considering MLIV in the differential diagnosis of patients in a pediatric age group with cerebral palsy-like presentation. Although the same rare mutation was seen in two apparently unrelated families, this was not seen in the sample screened from the general population. The HRM assay provides a cost-effective assay for population screening for the c.237+5G>A mutation.

## 1. Introduction

Defects in lysosomal degradation pathways result in a group of inherited disorders known as lysosomal storage disorders (LSDs). A deficiency of some lysosomal hydrolases sometimes causes the accumulation of undigested accumulating complex lamellar and granular material composed of mucopolysaccharides and lipids. These complexes are detected in histopathological staining and electron microscopy as membranous cytoplasmic inclusions and zebra bodies [1]. Mucolipidosis is categorized into four types: type I, type II, type III and type IV. Type IV mucolipidosis (MLIV) is a rare progressive neurodegenerative disorder causing severe psychomotor developmental delay, vision impairment due to progressive retinal degeneration and corneal clouding and achlorhydria. Mucolipidosis IV is usually underdiagnosed as it displays clinical heterogeneity and may present with non-specific symptoms and signs. Diagnosis is confirmed by documenting the presence of high gastrin level, the presence of thin corpus callosum in magnetic resonance imaging (MRI), accumulated amphiphilic lipids and water-soluble substances in skin biopsy and mutations in the *MCOLN1* gene [2]. Achlorhydria leads to iron deficiency anemia, a feature that is observed in some patients with MLIV.

MLIV is highly prevalent in the Ashkenazi Jewish (AJ) population, with only 20–30% of the reported patients seen in non-AJ populations [2]. The founder MLIV mutations in AJ (95%) are either c.416-2A>G: a splice site mutation in intron 3 (72% of patients) or c.1_788del: a deletion mutation including exons 1 to 7 (23% of patients) [2]. 

Middle East populations have relatively high consanguinity rates. The estimated prevalence of inborn errors of metabolism in Oman is 1 in 1555 live births, with LSDs accounting for 21–25% of the total number of patients [3,4]. This study reported a total of six patients with some mucolipidosis. We hereby report the clinical and molecular characterization of six patients with MLIV in two unrelated Omani families with a novel mutation in the *MCOLN1* gene. Given the novelty of the c.237+5G>A mutation and the rarity of MLIV in the general population, the mutation was hypothesized to be a founder mutation in the Omani population. If proven to be true, this would likely have a significant impact on the allele frequency in the general population. Driven by this hypothesis, the current study also aimed to screen control samples from the same geographical region for the *MCOLN1* mutation and assess the mutation age in the population.

## 2. Materials and Methods

### 2.1. Patient Enrolment and Clinical Characterization

Patients with genetic defects of *MCOLN1* were enrolled from the Sultan Qaboos University hospital in Oman. The patients were evaluated by clinical geneticists, ophthalmologists and pediatric neurologists. Enrolled patients had clinical manifestations consistent with the diagnosis of MLIV, and they were found to have elevated serum gastrin levels. All patients were examined by a certified medical geneticist with all standard dysmorphology examinations. Microcephaly was defined with head circumference below -2 standard deviations. Joint contractures were clinically evaluated by assessing the impact on the range of motion. To determine retinal function, electroretinography (ERG, LKC Technologies, Gaithersburg, MD, USA) was performed according to standard testing protocols recommended by the International Society for Clinical Electrophysiology of Vision (ISCEV).

### 2.2. Confirmation of MCOLN1 Mutation by DNA SANGER Sequencing

The *MCOLN1* gene was analyzed by PCR and sequencing of both DNA strands of the entire coding region and the highly conserved exon-intron splice junctions. Sequencing was performed by a service provider in some patients and confirmed the presence of homozygous c.237+5G>A mutation in intron 2 of *MCOLN1* gene at genomic position ch19: 7,525,171 (GRCh38/hg38 build). We performed a polymerase chain reaction (PCR) to confirm the presence of this mutation in remaining patients and family members. PCR was performed on genomic DNA using illustra™ PuReTaq™ Ready-To-Go™ PCR (GE Healthcare UK Limited, Hatfield, UK). Following primers were designed to flank the mutation site: forward: GAGTCCCTGCGACAAGTTTC, reverse: GTCTTGAACTCCCAGCCTCA. PCR products were further directed for DNA sequencing using a BigDye® Terminator v3.1 Cycle Sequencing Kit and Applied Biosystem Genetic Analyzer 3100, Applied Biosystem, (Foster City, CA, USA). Sequencing was performed in both forward and reverse strands using each of the PCR primers. 

### 2.3. Mutation Screening in the General Population

The two families with the c.237+5 G>A mutations originated from one region in Oman. The derived mutation is novel and has not been reported earlier, suggesting the presence of founder effect in the region. Therefore, we designed a new, high-resolution melting (HRM) assay to screen 1280 population samples from departmental research depositories [5,6]. The following primers were used in the MeltDoctor (Applied Biosystem, USA) HRM assay: HRM_forward: GATGCTGCAAGTG-GTCAAGA and HRM_reverse: TCTCTTCTTGG-AGC ACAGCA. The reactions were run in 7500 Fast Real-Time PCR System (Applied Biosystem, USA), and data were analyzed in HRM analysis software (v 2.0.2). The derivative melt curve and difference curve plots were used to call the genotype of the unknown samples. 

## 3. Results

### 3.1. Clinical Description of Patients in Two Families

**Figure 1:**
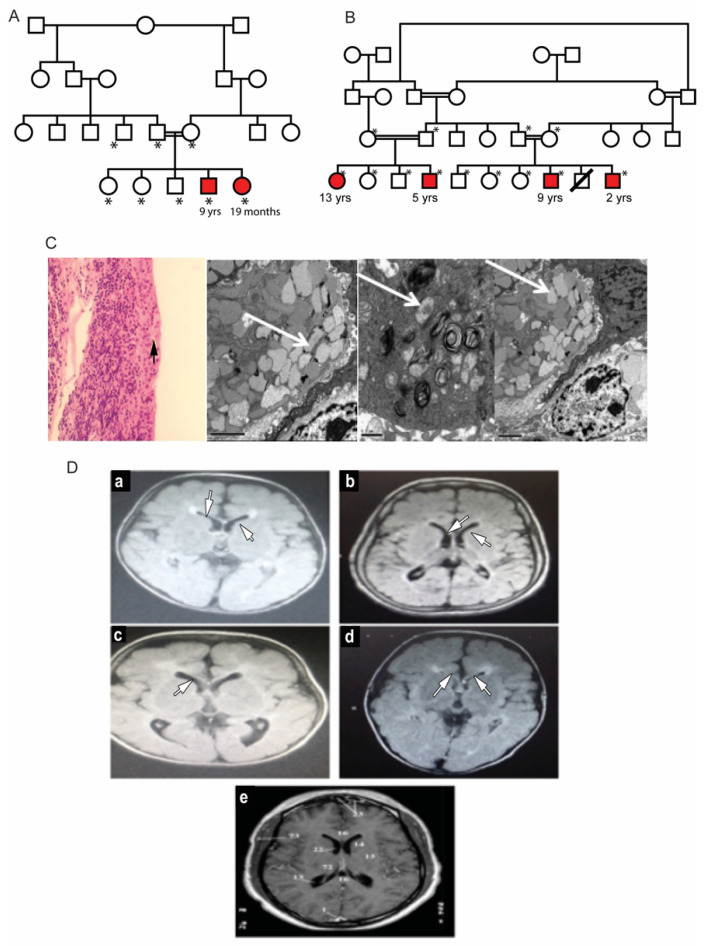
Clinical features of patients with mucolipidosis type IV. (**A**,**B**): Family pedigrees of two MLIV families. Affected siblings are highlighted in red color. *: individuals with an available DNA sample for molecular testing. (**C**): Conjunctival biopsy histological results. Left: H and E staining showing mild engorgement of goblet cells with secretions (black arrow). Right: Intra-cytoplasmic inclusion bodies in conjunctival tissue of MLIV patients. The bodies are multiple cytoplasmic single membranes with limited vacuoles (marked with white arrows). (**D**): Brain MRI findings in the studied MLIV patients. (**a**): Brain of the 9-year-old affected male of Family 1. (**b**–**d**): Patients of Family 2 aged 13, 5 and 9 years old, respectively. MRI images show hypomyelination and thinning of the corpus callosum (indicated by white arrows) with midline cerebellar atrophy. (**e**): A normal MRI of a 6-year-old brain for comparison.

Two unrelated families with multiple affected children were identified. The parents in both families were first cousins and were themselves unaffected (Figure 1A,B). In Family A, the proband, a 19-month-old girl (patient #2) was referred for evaluation of hypertonia and developmental delay. Her nine-year-old brother (patient #1) also presented with a developmental delay with poor vision and nystagmus since infancy. He was diagnosed and followed up as a case of hereditary spastic paraplegia. Dysmorphic features were similar in both patients, presenting with high nasal bridge, low hanging columella, low set ears, long, smooth philtrum, thin upper lips and widely spaced teeth. The ophthalmic evaluation of both patients revealed corneal haze. The dilated fundus examination in both children was difficult due to the corneal haze and showed optic disc pallor and retinal arteriolar attenuation. Electroretinography was performed in patient #1 and showed severe rod-cone dysfunction. Additional clinical details are described in Table 1. Laboratory investigations showed elevated blood gastrin levels. Both patients had iron deficiency anemia (Table 1). The conjunctival biopsy showed mild engorgement of goblet cells, and electron microscopy illustrated histological lipid storage particles and typical intracytoplasmic inclusion on electron microscopy (Figure 1C). 

**Figure 2:**
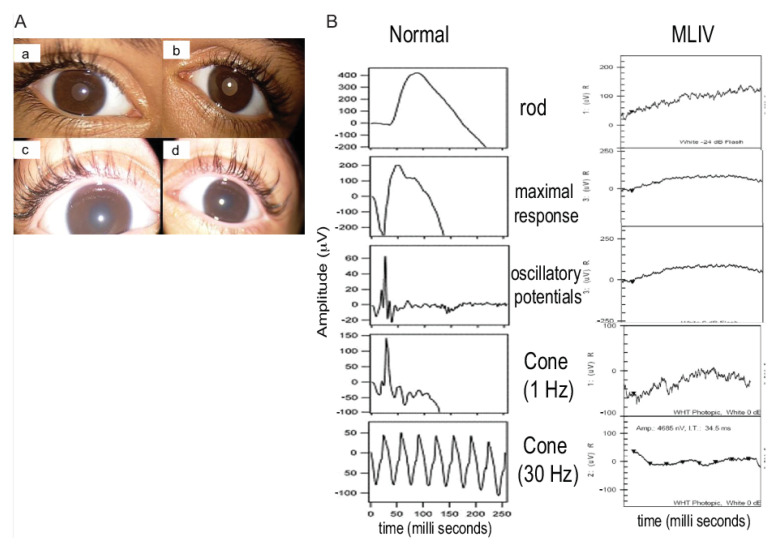
Ophthalmological findings in patients with mucolipidosis type IV (MLIV). (**A**): Anterior segment pictures showing mild corneal haze in the right (**a**) and left eye (**b**) in the 9-year-old patient of Family 2 (patient #5). Severe corneal haze in the right (**c**) and left (**d**) eyes of the 2-year-old patient of Family 2 (patient #6). (**B**): Full-field electroretinography (ERG) was performed using the LKC machine, according to the International Society for Clinical Electrophysiology of Vision (ISCEV) protocol. The electroretinogram of patient #6 of Family 2 is shown. All rod and cone response curves were flattened in the MLIV patient (right) compared to those obtained from a normal subject (left). The electroretinograms of other patients (1,3,4,5) were also severely reduced.

The second family, unrelated to Family 1, had four affected siblings aged between 2 and 13 years of age (Figure 1B). The proband, patient #3, was a 13-year-old girl who was initially diagnosed with spastic quadriplegia, with MRI findings of diminished brain parenchyma and atrophy of the corpus callosum (Figure 1D). Parents reported similar clinical manifestations in their five-year-old son (patient #4) and in two paternal cousins (patients #5 and #6, respectively). Some of the dysmorphic features in the four patients were bilateral ptosis, hazy cornea, prominent nasal tip, low set ears, long smooth philtrum and widely spaced teeth. The 13-year-old girl had a coarse face with thick eyebrows, while the other three patients had curly thick hair with receding hairlines. A large mouth was seen in all patients, except for the 13-year-old patient. An MRI of these patients’ brains showed mild cerebral atrophy with hypoplasia of the corpus callosum, with periventricular white matter changes. The patients showed different degrees of corneal haze and retinal abnormalities (Figure 2A). Additional clinical details are described in Table 1. Similar to Family 1, the patients had elevated fasting blood gastrin levels and iron deficiency anemia (Table 1). Conjunctival biopsy and findings from the histopathological evaluations were consistent with the diagnosis of MLIV. 

Rod and cones-function was assessed in all patients except one (the 19-month-old child in Family 1). Flattened waves were observed in all, indicating severe photoreceptor dysfunction (Figure 2B).

### 3.2. Mutation Analysis of MCOLN1 Gene and Population Screening

**Figure 3:**
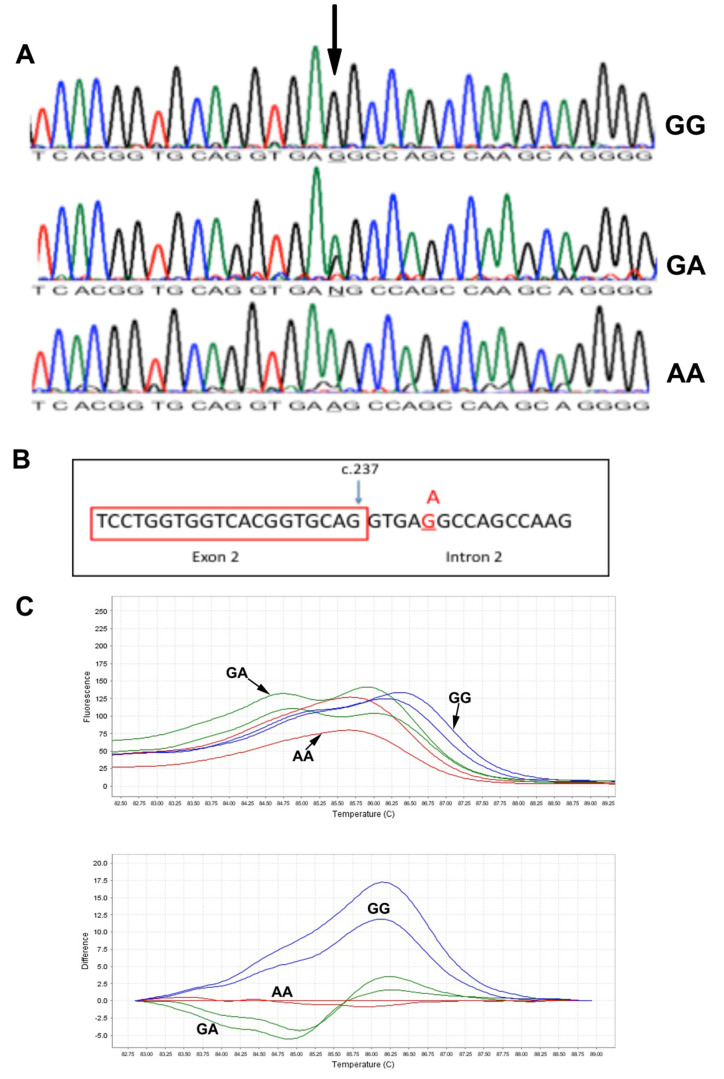
Molecular analysis of the *MCOLN1* c.237+5G>A mutation. (**A**): DNA Sanger sequencing results in subjects with GG, GA and AA genotypes. (**B**): Schematic presentation of the c.237+5G>A mutation at the junction of exon 2 and intron 2. (**C**): High-resolution melting results: melting derivative plot (upper) and difference plot (lower). GG: blue line, GA: green lines, AA: red lines.

DNA sequencing results of all patients showed a splice variant in intron 2 of the *MCOLN1* gene c.237+5 G>A (Figure 3A). The mutation was segregated in the two families. We further predicted the effect of c.237+5 G>A mutation on gene splicing activity using three algorithms: maximum entropy model (MAXENTSCAN and MAXENT), Maximum Dependence Decomposition (MDD), First-order Markov Model (MM) and eight Matrix Model (WMM) [7]. All tested algorithms resulted in a reduced splicing score when the mutation site contains an A nucleotide (Figure 3B), suggesting a pathogenic nature of the mutation, Table 2. This will retain intron 2 (1272 bp) in the *MCOLN1* transcript, which may result in longer mRNA. Similar to intron 2 being retained, a premature stop codon will be introduced. The stop codon UGA starts at base 58 of intron 1 of the *MCOLN1* gene. The predicted protein size of the mutated version will be around 10 kDa (98 amino acids), which could be detected in Western blotting. 

### 3.3. c.237+5 G>A Analysis with High-Resolution Melting and Determination of Founder Effect

The two unrelated families with the c.237+5 G>A mutations came from the same geographical region in Oman. The mutation is a novel mutation not being reported in the SNP database (dbSNP), Exac and Greater Middle East Variome. We designed an HRM assay to screen larger sets of samples from the population. In the melting derivative plot, subjects with the GG genotype (blue lines) show a higher melting temperature with peaks shifted to the right (Figure 3C). Subjects with AA genotypes (red lines) have lower melting temperatures compared to GG genotypes. Subjects with GA genotypes (green line) show a double peak line (Figure 3C). We could not detect any heterozygous sample in this set and could not perform mutation age analysis. 

## 4. Discussion

MLIV is an autosomal recessive disease caused by mutations in the *MCOLN1* gene characterized by progressive neuro-ophthalmic manifestations. MLIV is a multi-ethnic disease but has been mostly reported in the Ashkenazi Jewish population. However, other patients were reported in non-Jews, including Italian, Indian and Turkish populations [8,9,10].

We document the manifestations of MLIV in multiple members of two Omani families. A novel mutation was detected in the two families at the splice acceptor site c.237+5G>A. An HRM assay for general population screening and future cascade screening in extended family members was established. 

More than twenty causative mutations in MCOLN1have been described as underlying MLIV. The majority are deletion or splice site changes [2]. The reported c.237+5G>A in Oman is a novel mutation in a unique population with a high rate of consanguineous marriages.

Most of the patients described in this study were first diagnosed with spastic paraplegia. The neurological manifestations of MLIV mimic other disorders with similar neurological manifestations, contributing to frequent misdiagnosis [9]. Corneal clouding is considered a classical and diagnostic sign of MLIV but may be absent in some patients [9]. Furthermore, MLIV could clinically present as a mild or late-onset disease, which may also lead to a mistaken diagnosis [11]. There are a large number of patients being followed up in pediatric departments with a diagnosis of cerebral palsy and neurodegenerative disease without thorough investigations. 

Given the rarity of the c.237+5G>A mutation and high background consanguinity rates in Oman, this mutation could represent a founder effect, at least in the region where the two families come from. Although heterozygous carrier in the screened control samples was not identified, we cannot exclude that the control population is not representative of the small tribal or geographical region. 

## 5. Conclusions

We report MLIV in two families from the Middle East with a novel mutation. The current study stresses the importance of molecular diagnosis and considers testing for disorders that may otherwise be of higher representation in other ethnic groups. The outcome of this research will be useful for the MLIV patients enrolled in the study and their families, as it will help them in future of starting families and having children.

## Figures and Tables

**Table 1:** Clinical features of patients with mucolipidosis type IV in this study.

	Family 1		Family 2			
Patient #	1	2	3	4	5	6
Age	9 yrs	19 months	13 yrs	5 yrs	9 yrs	2 yrs
infantile onset of global D.D	+	+	+	+	+	+
Spastic quadriplegia	+	+	+	+	+	+
Contractures	+	−	−	−	+	+
Microcephaly	+	−	+	+	+	+
Dysmorphism	+	+	+	+	+	+
**Blood Biochemistry:**						
Iron (11–28 μmol/L)	2	5	2	4	2	ND
Ferritin (24–336 ng/mL)	2	5	2	5	2	ND
Hemoglobin (11–15 g/dL)	6.1	104	7	9	9.4	6.9
Gastrin (15–110 ng/L)	706	1691	1474	ND	801	ND
**Ophthalmological findings:**						
Photophobia	−	−	+	−	+	+
Nystagmus	+	+	−	−	−	−
Strabismus	RX	RE	AXT	RE	LX	LX
Corneal haziness	+	++	+++	+++	+++	+++ (Rt) ++ (Lt)
Pupil reaction to light	sluggish	sluggish	N	sluggish	N	sluggish
Pigmentary retinopathy	+	+	No view	+	No view	No view
ERG- rod cone dysfunction	+++	ND	+++	+++	+++	+++
**Brian MRI:**						
Thin corpus callosum	+	+	+	+	+	+
Periventricular white matter changes	+	+	+	+	+	+

Values in brackets are normal ranges. D.D: Developmental delay. +: present, N: Normal, ND: not done. Rt: right eye, Lt: left eye. RX: right exotropia. LX: left exotropia. AXT: alternating exotropia. RE: right esotropia. −: not present; +: mild; ++: moderate, +++: severe.

**Table 2:** 5′ splice site prediction model for the MLIV c.237+5 G>A mutation.

Splice Site	Sequence	MAXENT	MDD	MM	WMM
wild-type	cag*GTGAGG	10.07	13.38	10.18	10.76
Patient	cag*GTGAAG	6.66	11.18	7.41	7.31

Mutation site is underlined. *: exon-intron junction. MAXENT: maximum entropy model, MDD: Maximum Dependence Decomposition, MM: First-order Markov Model. WMW: eight Matrix Model.

## Data Availability

Not applicable.
